# Protocol for the study of self-perceived psychological and emotional well-being of young Paralympic athletes

**DOI:** 10.1186/s12955-017-0798-2

**Published:** 2017-11-13

**Authors:** Luca Puce, Lucio Marinelli, Laura Mori, Ilaria Pallecchi, Carlo Trompetto

**Affiliations:** 1 Department of Neuroscience, Rehabilitation, Ophthalmology, Genetics, Maternal and Child Health, University of Genoa, Genoa, Italy; 2 CNR-SPIN, Physics Department, University of Genoa, Genoa, Italy

**Keywords:** Psychophysical well-being, Young disabled athletes, Paralympic sport

## Abstract

**Background:**

We present the detailed protocol set up to investigate how agonistic sport affects the self-perceived psychological and emotional well-being of disabled young people.

**Methods:**

The study will be carried out on a number of subjects as large as 800–1200, using well-established indices that give a quantitative measure of such well-being, namely SF-12 and PGWBI. The related questionnaires will be administered to the participants to a forthcoming international event, the European Para-Youth Games, 9–15 October 2017, Liguria, Italy, as well as to a reference population of a similar number of subjects, made up of young disabled people that do not practice agonistic sport.

**Discussion:**

We expect that the outcomes of the study may strongly impact not only the socio-sanitary field but also society in general, as disabled people can be considered an extreme situation in the issue of balancing individual needs and environment to pursue psychological well-being.

**Trial registration:**

ISRCTN14389453 (29 June 2017).

## Background

The dynamic balance between personal needs and potentiality on one hand and the external environment on the other hand defines the psychological well-being of an individual. [1] The achievement of a high level of psychological well-being is a primary target for any human being. In the framework of the current scientific theories, psychological well-being can be viewed as a multifaceted gem, in that a variety of aspects are contributory to its enhancement [2–5]. Among these, self-acceptance, strong and lasting affection bonds and independence are clearly crucial, but likewise important is the willingness of shaping our own life as a steady growth toward the realization of objectives that may add value to our own existence. In the case of disabled people, the natural unbalance of needs/potentialities and environment makes even more urgent to pursue the enhancement of psychological well-being, addressing the integration in a positive and gratifying context that could promote personal evolution. Sport activities, especially at agonistic level, are likely the most effective means for the practical realization of this strategy [6, 7]. Agonistic sport helps disabled people to grow, take responsibilities, succeed, as well as fail, in other words to learn how to face the challenges of life. This is particularly crucial in a stage of life - adolescence and young adulthood - when major mental and physical changes occur, making the effect of positive and negative external inputs even more critical. [8, 9] In literature, few studies address the effect of agonistic sport on the psychological well-being, social competence and self-concept, yet they are limited to specific contests, either in terms of kind of syndrome [10, 11], kind of practiced sport [12–16], geographical origin and number of subjects under examination [12–14]. The International Paralympic Committee (IPC) is responsible for the organization, coordination and supervision of international sport events and its mission is offering disabled athletes of whichever level the opportunity to achieve excellence in sports, at the same time fostering such values as courage, determination, motivation and equality [17]. Whether or not this mission is effectively transferred in actual facts, yielding a real improvement of the *psychophysical and emotional well-being of disable athletes* has not yet been investigated so far, *over large numbers*, by a scientific approach. Hence there may exist a gap between the programmatic abstractness of the values promoted by IPC and the actual effectiveness, assessed by suitable quantitative parameters, of the sport activities organized and managed by IPC itself, in terms of overall psychological well-being, physical and mental health, emotional state.

General consensus is currently shared by the scientific community about the scope, potential and limits of investigation methods based on the *self-perceived assessment of health*. The diffusion of such methods has triggered the process toward the necessary standardization and real conditions validation of criteria and procedures, which in turns guarantees a high level of reliability in a variety of contexts. Several quantitative indices based on self-perceived health, either syndrome-specific or generic, have been developed since the sixties [18, 19]. The generic indices have been applied to general and patient populations in a number of different contexts such as the assessment of clinical trials, impact of medical, surgical and pharmacological therapies, palliative treatments, health care practices, impact of environmental factors on people, post-discharge from hospital condition, syndromes that directly or indirectly affect the psychological well-being [20–23]. Whereas physical well-being is strictly related to the type and severity of pathologies, psychological well-being may not be necessarily closely dependent on it, which accounts for the variety different contexts in which psychological well-being indices have been used in.

In our project, we plan to survey and analyze the self-perceived psychophysical, physical, emotional well-being of young age disabled Paralympic athletes taking part to the European Para-Youth Games, 9–15 October 2017, Liguria, Italy. The results will be compared with the ones obtained on a population sample composed of young age disabled subjects that do not practice sport at agonistic level. The outcome of our study will be instrumental to the assessment of the effect on individual well-being of agonistic sport and related events that foster self-motivation, self-fulfillment, and social aggregation.

Given the large number of subjects to be examined, it is crucial to set up a well-structured and detailed protocol that guarantees a high level of homogeneity in the administration methods, and thus reliability of the outcomes. This is the object of the present paper.

## Methods/design

### 2.A. Tools

In this study, we choose indices that measure the psychological well-being, on the basis of the following issues:

*(i)* focus on the psychological and emotional well-being, rather than on the physical well-being. This is a key requirement when probing a significantly heterogeneous sample of subjects having different types of syndromes or disability. Indeed, the physical well-being and ease in carrying out regular daily activities is dramatically dependent on the specific disability, which would invalidate the comparison of results on different subjects, while the psychological and emotional well-being, which relies rather on self-motivation and perspectives, can be considered beyond any specific type of syndrome and social context,

*(ii)* generic (not syndrome-specific) character, which makes the indices suitable for different types and levels of disability.

*(iii)* subdivision in multiple domains, which allows a more structured data analysis that could finally trace a health and psychophysical/emotional profile.

*(iv)* short time required to fill-in the questionnaires; this is a relevant requirement when dealing with a large number of subjects.

*(v)* possibility of being either self-administered or administered as a face-to-face interview, the latter being suitable for subjects that are either visually impaired, with mental disability, not speaking the English language or illiterate.

*(vi)* widespread and documented utilization.

On the basis of the above mentioned criteria, we use two indices that provide a quantitative probe of self-perceived psychophysical and emotional well-being, namely the Psychological General Well-Being Index (PGWBI) [24] and the Short Form SF-12 index. [25] The former index is based on a questionnaire made up of 22 items selected from longer previous versions, originally developed in the US by the same authors and later validated in several European countries, with corresponding different language versions available on the website of the Mapi Health Research & Commercialization institute [26]. It can be either self-administered or administered as an interview. Each one of the 22 items has six possible answers, whose score is proportional to the level of psychological and general well-being, rated on a six-point scale from 0 to 5. The 22 items are sorted in six different domains, namely anxiety, depressed mood, positive well-being, self-control, general health and vitality. Each domain is defined by a minimum of 3 to a maximum of 5 items. The scores for all domains can be summed up to provide the total score, which reaches a maximum of 110 points, representing the best achievable well-being. The questionnaire structure is well balanced with items that are presented *(i)* either as questions or as statements, *(ii)* either with increasing or decreasing score from the first to the last option, *(iii)* either addressing intensity or frequency of a certain occurrence. It takes no longer than 20 min to fill-in. The PGWBI index is among the most used ones in clinical research projects and its diffusion is such that it is often used as a reference tool for the development or validation of other new tools.

The second index that we will use is the Short Form-12 (SF-12) [25], derived from the original longer version SF-36 [27] and having a two-fold character. Indeed, it addresses two different aspects of health, namely physical health and mental health, that are measured by two synthetic indices, PCS-12 (Physical Component Summary) and MCS-12 (Mental Component Summary), reflecting a combination of physical and mental function and well-being, the degree of disability on social and individual basis and a personal evaluation of general health. In this work we are interested in mental health, while physical health will be treated as ancillary information in the data analysis. Likewise PGWBI, it is thought to be either self-administered or administered as an interview. It is made up of 12 items and assesses eight domains. Four out of the eight domains (physical functioning, role limitations due to physical health, role limitations due to mental health, emotional well-being) include two items, while the other four domains (bodily pain, vitality, social functioning, general health) include a single item. The scores PCS-12 and MCS-12 are calculated as weighed sums of the given answers to the items related to physical health and mental health, respectively, using tabulated weighs and additive constants obtained from the regression of data collected from an American population sample [28, 29]. In the algorithm for the computation of the scores, before summing up, the most positive answers to each item are systematically rejected in any case, regardless they have been chosen or not. In the questionnaire, the subject is asked how he/she feels like and how he/she manages to carry out daily activities, considering the four-week time interval just culminating in the day that the questionnaire is filled-in. Filling-in takes no longer than 10 min.

The SF-12 widespread use is due to its favorable ratio of brevity to reliability. The combined use of PGWBI and SF-12 indices is recommended [30].

**Fig. 1:**
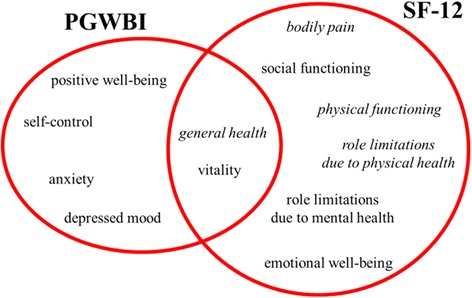
Sketch of the domains addressed by the PGWBI and SF-12 indices, showing that two of them are shared by the two indices. In italic font are the domains pertaining to physical health, while in non-italic font are the ones pertaining to mental health

The domains addressed by the two indices are sketched in Fig. 1, where it can be seen that two of them (general health, vitality) are shared by both indices.

In appendices C and D, questionnaires of the two indices are reported textually in their original United States versions.

### 2.B subjects

The subjects under examination are the young disabled Paralympic athletes taking part to the European Para-Youth Games, 9–15 October 2017, Liguria, Italy. Disabled people aged 12 to 23 are eligible. This international event, taking place every two-three years, is hosted each time in a different European country. On the basis of the figures registered in the former editions in Croatia (223 athletes from 22 countries) and Czech Republic (347 athletes from 14 countries) and in other similar international events, we expect an even larger number of participants to the forthcoming edition. Accordingly, in the official press conference of the event (held in Genoa, Italy, on April the 5th, 2017 [31]) 800 to 1200 participants coming from 27 countries (Belgium, Bulgaria, Croatia, Denmark, Estonia, Finland, France, Germany, United Kingdom, Greece, Israel, Italy, Lithuania, Luxemburg, Montenegro, The Netherlands, Norway, Portugal, Czech Republic, Romania, Serbia, Slovakia, Slovenia, Spain, Sweden, Turkey and Hungary) were announced. These figures could even be larger in the case that the IPC re-admits Russian athletes, formerly banned due to the case of the state doping in 2016 [32]. We expect that most of the participants take part to our survey, indeed, to encourage the participation, expressing the willingness or unwillingness to fill-in the questionnaires will be set as a mandatory prerequisite for the participation to the scheduled competitions.

The questionnaires will be also delivered to similar numbers of young disabled people that do not practice agonistic sport, which will work as control groups. These reference subjects will be selected to be distributed within the same age range, countries, levels of education and socio-economic conditions as the sample of disabled Paralympic athletes under study.

As exclusion criterion, we will consider not eligible those subjects that are affected by serious intellectual disability, which are not able to assess the self-perceived well-being objectively.

### 2.C description of the event

**Fig. 2:**
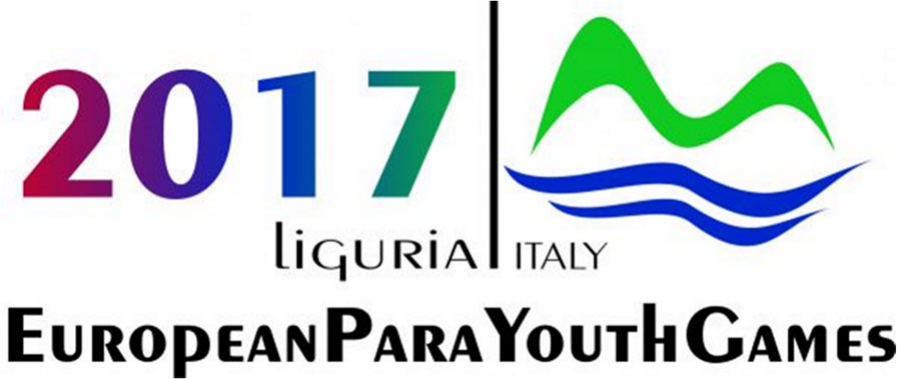
Logo of the European Para-Youth Games, 9–15 October 2017, Liguria, Italy

Liguria, Italy, will host the third edition of the European Para-Youth Games, 9–15 October 2017 (official webpage www.epyg2017.com). In Fig. 2, the logo of the event is shown. The time schedule of the event is the following:

- October 11: opening ceremony.
- October 9–11: arrivals of the athletes; medical examinations for the assignment of classifications [33], necessary to participate in the competitions; trainings.
- October 12–14: competitions.
- October 14: closing ceremony.
- October 15: departures of the athletes.

Competitions in as many as eleven sports will be organized, subdivided into basic sports (athletics, swimming, table tennis, boccia), other Paralympic sports (football 5-a-side, football 7-a-side, goalball, judo, sailing, sitting volleyball) and demonstration non-competitive sports (archery).

Each of these sports has its own technical regulations and its own federation. The age range for eligibility to the event slightly differs from sport to sport, however always within the range 12 to 23.

The organizing committee and the president and marketing and communication delegate have approved the present research project, giving exclusive rights to the authors.

The principal investigator of this project (L.P.) has been appointed by the FINP (Italian Federation for Paralympic Swimming) to be technical director of the event. This role will ease the set-up, management and funding of the present project in all its practical aspects.

### 2.D protocol of the experimental procedure

The questionnaires will be administered in their original English language versions, in anonymous form, in four different modes:

- self-administered via web
- self-administered in paper format
- face-to-face interview with the assistance of someone else, via web
- face-to-face interview with the help of a tutor or a linguistic mediator, in paper format

One month prior to the event, notification and information about the questionnaires will be posted on the official web page of the event itself and sent via e-mail to the federations of various sports. A link in the event web page will redirect to a web form developed by Google Drive package, containing an explanatory note (see appendix A), a form where the subjects will be asked to express either their willingness or unwillingness to take part in the survey (see appendix B), the questionnaires to be filled-in (see appendices C and D), an additional questionnaire containing auxiliary information (see appendix E). The explanatory note describes the main characteristics and aims of the present study and the anonymous and voluntary nature of the participation. The ticked willingness/unwillingness choice, in case of positive answer (willingness to participate), gives the subject access to the two questionnaires (appendices C and D), plus the additional questionnaire which contains auxiliary information to be used for articulated data analyses. Specifically, in this questionnaire - which likewise the other questionnaires is identical for the four administration modes and for the Paralympic and reference samples - questions about age, gender, country, level of education, practiced agonistic sport (if any), IPC disability class (if any), living setting (either rural or urban area), kind of disability (if any), how the questionnaires have been filled-in (either self-administered or with the help of someone else in the case of web submission, specifying the entity of the help; self-administered or as a face-to-face interview with the help of a tutor or a linguistic mediator in the case of paper format fill-in, specifying the entity of the help), place where the questionnaires have been filled-in (appendix E). Primary outcomes of this study are the psychological well-being measured by the PGWBI index and the emotional well-being measured by the MCS-12 index of Short Form SF-12. Secondary outcomes are the physical well-being measured by the PCS-12 index of Short Form SF-12 and demographic information on the subjects under examination (gender, age, country, type of disability) obtained from the additional questionnaire.

A printed evidence (hard paper copy or pdf file) of the page with the ticked willingness/unwillingness choice will have to be produced by the Paralympic athletes to the organizers of the event at the time of accreditation to the competitions. At the time and venue of the event, specifically at the time of the medical examinations for the assignment of classifications prior to the competitions, those athletes which have not yet expressed their willingness/unwillingness to participate in the survey will be handled the three questionnaires, plus the form to express willingness/unwillingness to participate, in paper format.

Considering each individual case, the questionnaires will be self-administered or administered as a face-to-face interview with the help of a tutor or a linguistic mediator. Both these roles will be played by volunteers among the 1000 expected ones, chosen by the organizers of the event among high school and university students of foreign languages. Tutors or a linguistic mediators should encourage participants to fill-in the questionnaires, solve doubts, make sure that the questions are understood and answers are given with awareness, check correctness and completeness of the procedure. Moreover, they will be given detailed instructions for the administration of questionnaires, according to the suggestions and recommendations of the respective authors of the questionnaires. In particular, among other things, they should present and describe the questionnaires according to given guidelines, have a friendly and welcoming attitude, repeat questions more times if necessary, be thankful at the end. On the other hand, they should avoid talking explicitly of the disability of the subject, force the subject to answer, give their own interpretation of the questions, accept incomplete questionnaires, underrate the importance of the questionnaires. Also guidelines for specific occurrences will be given, such as in the cases that the participant refuses to answer, handles incomplete questionnaires, asks for further explanations, raises an issue with privacy. The strict observation of these instructions, joined to the effort of making as homogeneous as possible the conditions and modes of administration of the questionnaires throughout the participants, will contribute to the reliability of the outcomes.

Roughly, we expect that 70% of the athletes will use the web submission form in advance of the event, using personal computers or smartphones, while another 30% will fill-in the paper questionnaires at the medical classification. The remaining athletes (for example those who have already been assigned a disability class and do not need to pass the classification step) will be asked to fill-in the questionnaires and willingness/unwillingness form at the moment of the accreditation to the competitions. By this procedure, all the athletes will fill-in the questionnaires prior to the competitions, so that any influence of the positive or negative performance in the competitions on the mind attitude while filling-in the questionnaires will be ruled out. The expression of willingness/unwillingness will be set as a mandatory requisite for the accreditation to the competitions. The fact that the principal investigator of this work is in the organization and technical staff of the event will help implementing this constraint.

Around one month after the event, the same questionnaires and explanatory note will be administered to a reference sample of young disabled people that do not practice agonistic sport. In this case, the web submission route accessible via the official site of the event will be used exclusively. To reach the targeted number of young disabled people (nearly the same as the number of young Paralympic athletes), physiatric clinics across European countries taking part into the event will be contacted. In these structures, contact people will be identified within the personnel and given instructions for administration of the questionnaires and assistance, similarly as tutors and linguistic mediators. They will choose for each individual case, whether to use self-administration or face-to-face interview procedures.

Tentatively, we foresee to complete data collection within the following three months since the time of the event.

### 2.E data analysis

As a first step, data in paper form will be digitalized and all the data will be collected in a single digital database, whence scores related to the PGWBI and SF-12 indices will be computed. Normal distribution of the scores of both questionnaires will be inspected both visually and using Shapiro-Wilks test. Outlayer scores deviating more than 3 standard deviations from the mean value will be rejected. Also correlation checks recommended by the authors of the questionnaires [28, 29] will be carried out. Finally, the results of the young Paralympic athlete sample will be compared with those of the reference sample of young disabled people that do not practice agonistic sport using Kruskal-Wallis test for independent samples. Beside the comparisons over the whole samples, more articulated analyses will be carried out by restricting the comparison to specific sub-groups of subjects (type of disability, country, age, etc.). In addition, well-being profiles will be drawn through analyses focused on one or more selected domains, out of the twelve ones covered by the PGWBI and SF-12 indices, particularly those domains that are more relevant to the values promoted by the IPC. The correlation between the IPC disability class, which is a measure of the disability level of Paralympic athletes, and the well-being score will be studied, in order to investigate whether higher scores can be obtained also by athletes affected by serious disabilities. Finally, a number of subjects will be identified in the reference sample, among people that practice sport on an individual self-managed basis, not at agonistic level. The comparison of results from this group with the ones of the Paralympic athletes will give clues on the factors that affect mostly the psychological well-being, namely the physical practice of sports on one hand and of the aspects of motivation, social aggregation and self-fulfillment promoted by the IPC activities on the other hand. Furthermore, insight will be pursued on whether any improvement of the self-perceived well-being is primarily of psychological origin (and possibly also physical, as a consequence) or else of physical origin (and possibly psychological, as a consequence).

## Discussion

### 3.A dissemination

As for dissemination, the close relationship of this project with an international event that will be given very broad media coverage will provide a natural dissemination channel through the media themselves. Furthermore, the principal investigator of this project being part of the organization staff of the event will further enhance the dissemination potential of this close relationship.

A press conference of announcement of the event has already taken place [31] and therein the forthcoming collaboration of the organization of the event with the institution of the authors of the present work (University of Genoa) was pointed out. Similarly, articles appeared on general-scope newspapers and news sites have mentioned this collaboration. It is plausible to assume that this represents the beginning of a significant media coverage from mainstream news channels, through press releases. Moreover, information about this project will be conveyed not only on the official web site of the event but also on the web sites of all the involved organizations (International Paralympic Committee IPC, Italian Paralympic Committee CIP, Italian Federation of Paralympic Swimming FINP, Italian Federation of Paralympic and Experimental Sports FISPES, Italian Federation of Paralympic Sport for Intellectual and Relational Disabilities FISDIR, Italian Federation of Paralympic Sport for visually impairment disabilities FISPIC, Italian Federation of Archery FITARCO, Italian Federation of Sailing FIV, Italian Federation of Table Tennis FITET). Finally dissemination via social networks will be also extensive (hashtag *#epyg2017*).

The results of this work will be also published on scientific medical journals whose mission is related to quality of life and sport and presented at scientific congresses. An oral presentation on this protocol has been scheduled at the 45th S.I.M.F.E.R. national congress (Genoa, Italy, October 22–25, 2017).

### 3.B expected results, impact and perspectives

From this study we expect an objective and scientific assessment of the effectiveness of the programmatic lines that guide the methods and activities of the IPC. In addition, the detailed analysis of results of selected domains, out of the twelve ones of the PGWBI and SF-12 indices, may even suggest specific operative indications to IPC itself on how to address in a more focused way the forthcoming activities or introduce procedural changes in the same activities, keeping the psychological and emotional well-being of disabled athletes as the primary objective. For example, we can envisage such things as the set-up of ceremonial protocols that could better fulfill the psychological needs of young athletes, the fostering of collateral aggregation opportunities and exchange events within competitions, the organization of initiatives that give voice and visibility to the athletes.

The results of this work have relevance both within the medical community and within society in general, with a transnational valence. Indeed, we believe that the psychological well-being of disabled people is a measure of the degree of civilization of a country. Hence this work will somehow assess the civilization of European counties and possibly trigger a process toward higher civilization which could make disabled people feel less handicapped.

Finally, the role of agonistic activities in personal motivation and growth of disabled people, especially of young age, may represent a model for able-bodied people as well.

### 3.C limitations of this study

This study will investigate the European context, but we are aware that different results could be obtained in other parts of the world, especially in eastern Asia countries. In addition, further limitations may be identified a posteriori, in the case that the Paralympic and reference samples are significantly unbalanced in terms of gender, age, type of disability or mode of administration of the questionnaires.

## Conclusions

This manuscript presents the protocol designed to carry out a study of the self-perceived psychophysical, physical, emotional well-being of young age disabled people that practice sport at agonistic level. Two well-established indices that provide a quantitative probe of physical, psychophysical and emotional well-being will be used, namely the PGWBI and the SF-12 indices. The population sample of 800–1200 disabled subjects under examination will be recruited among Paralympic athletes taking part to the European Para-Youth Games, 9–15 October 2017, Liguria, Italy. The results will be compared with the ones obtained on a reference population sample composed of young age disabled subjects that do not practice sport at agonistic level. The data analysis will yield an assessment on the role of agonistic sport practice and related events in fostering self-motivation, self-fulfillment, and social aggregation of disabled people, thus providing results that could serve as guidelines for health care policies and for society in general, with a transnational valence.

## Appendix A

Explanatory note on the project

The attached questionnaires are well established and validated scientific tools, that will be used in the framework of a scientific project, whose goal is assessing the self-perceived psychological and emotional well-being of young Paralympic athletes as compared young disabled people that do not practice agonistic sports. The questionnaires have anonymous character, they are used only for statistical analysis and they do not require any longer than 30 min to fill-in.

## Appendix B

Having been informed about the ongoing study on self-perceived psychological and emotional well-being of young Paralympic athletes, herewith I express my (tick the appropriate box)

□ willingness

□ unwillingness

to fill-in, in anonymous and voluntary form, the attached questionnaires about the self-perceived psychological and emotional well-being and about my personal details.

For the young Paralympic athletes participating to the European Para-Youth Games, 2017, Liguria, Italy:

The willingness/unwillingness choice can be expressed either via this web form or in paper format at the venue and time of the event. Likewise, questionnaires can be filled-in either via this web form or in paper format at the venue and time of the event. Please, consider that the expression of willingness/unwillingness in either forms is a mandatory requisite for the participation to the scheduled competitions.

## Appendix C

Psychological General Well-Being Index (PGWBI) questionnaire [24]. Tick the appropriate box for each item.

1. How have you been feeling in general during the past month?

□ In excellent spirits

□ In very good spirits

□ In good spirits mostly

□ I have been up and down in spirits a lot

□ In low spirits mostly

□ In very low spirits

2. How often were you bothered by any illness, bodily disorder, aches or pains during the past month?

□ Every day

□ Almost every day

□ About half of the time

□ Now and then, but less than half the time

□ Rarely

□ None of the time

3. Did you feel depressed during the past month?

□ Yes – to the point that I felt like taking my life

□ Yes – to the point that I did not care about anything

□ Yes – very depressed almost every day

□ Yes – quite depressed several times

□ Yes – a little depressed now and then

□ No – never felt depressed at all

4. Have you been in firm control of your behavior, thoughts, emotions or feelings during the past month?

□ Yes, definitely so

□ Yes, for the most part

□ Generally so

□ Not too well

□ No, and I am somewhat disturbed

□ No, and I am very disturbed

5. Have you been bothered by nervousness or your “nerves” during the past month?

□ Extremely so – to the point where I could not work or take care of things

□ Very much so

□ Quite a bit

□ Some – enough to bother me

□ A little

□ Not at all

6. How much energy, pep, or vitality did you have or feel during the past month?

(check one box)

□ Very full of energy – lots of pep

□ Fairly energetic most of the time

□ My energy level varied quite a bit

□ Generally low in energy or pep

□ Very low in energy or pep most of the time

□ No energy or pep at all – I fell drained, sapped

7. I felt downhearted and blue during the past month.

□ None of this time

□ A little of the time

□ Some of the time

□ A good bit of the time

□ Most of the time

□ All of the time

8. Were you generally tense or did you feel any tension during the past month?

□ Yes, extremely tense, most or all of the time

□ Yes, very tense most of the time

□ Not generally tense, but did feel fairly tense several times

□ I felt a little tense a few time

□ My general tension level was quite low

□ I never felt tense or any tension at all

9. How happy, satisfied, or pleased have you been with your personal life during the past month?

□ Extremely happy – could not have been more satisfied or pleased

□ Very happy most of the time

□ Generally satisfied, pleased

□ Sometimes fairly happy, sometimes fairly unhappy

□ Generally dissatisfied or unhappy

□ Very dissatisfied or unhappy most or all the time

10. Did you feel healthy enough to carry out the things you like to do or had to do during the past month?

□ Yes – definitely so

□ For the most part

□ Health problems limited me in some important ways

□ I was only healthy enough to take care of myself

□ I needed some help in taking care of myself

□ I needed someone to help me with most or all the things I had to do

11. Have you felt sad, discouraged, hopeless, or had so many problems that you wondered if anything was worthwhile during the past month?

□ Extremely so – to the point that I have just about given up

□ Very much so

□ Quite a bit

□ Some – enough to bother me

□ A little bit

□ Not at all

12. I woke up feeling fresh and rested during the past month.

□ None of the time

□ A little of the time

□ Some of the time

□ A good bit of the time

□ Most of the time

□ All of the time

13. Have you been concerned, worried, or had any fears about your health during the past month?

□ Extremely so

□ Very much so

□ Quite a bit

□ Some, but not a lot

□ Practically never

□ Not at all

14. Have you had any reason to wonder if you were losing your mind, or losing control over the way you act, talk, think, feel or of your memory during the past month?

□ Not at all

□ Only a little

□ Some – but not enough to be concerned or worried about

□ Some and I have been little concerned

□ Some and I am quite concerned

□ Yes, very much so and I am very concerned

15. My daily life was full of things that were interesting to me during the past month.

□ None of the time

□ A little of the time

□ Some of the time

□ A good bit of the time

□ Most of the time

□ All of the time

16. Did you feel active, vigorous, or dull, sluggish during the past month?

□ Very active, vigorous every day

□ Mostly active, vigorous – never really dull, sluggish

□ Fairly active, vigorous – seldom dull, sluggish

□ Fairly dull, sluggish – seldom active, vigorous

□ Most dull, sluggish – never really active, vigorous

□ Very dull, sluggish, every day

17. Have you been anxious, worried, or upset during the past month?

□ Extremely so – to the point of being sick or almost sick

□ Very much so

□ Quite a bit

□ Some – enough to bother me

□ A little bit

□ Not at all

18. I was emotionally stable and sure of myself during the past month.

□ None of the time

□ A little of the time

□ Some of the time

□ A good bit of the time

□ Most of the time

□ All of the time

19. Did you feel relaxed, at case or high strung, tight, or keyed-up during the past month?

□ Felt relaxed and at ease the whole month

□ Felt relaxed and at ease most of the time

□ Generally felt relaxed but at times felt fairly high strung

□ Generally felt high strung but at times felt fairly relaxed

□ Felt high strung, tight, or keyed-up most of the time

□ Felt high strung, tight, or keyed-up the whole month

20. I felt cheerful, lighthearted during the past month.

□ None of the time

□ A little of the time

□ Some of the time

□ A good bit of the time

□ Most of the time

□ All of the time

21. I felt tired, worn out, used up, or exhausted during the past month.

□ None of the time

□ A little of the time

□ Some of the time

□ A good bit of the time

□ Most of the time

□ All of the time

22. Have you been under or felt you were under any strain, stress, or pressure during the past month?

□ Yes – almost more than I could bear or stand

□ Yes – quite a bit of pressure

□ Yes, some – more than usual

□ Yes, some – but about usual

□ Yes – a little

□ Not at all

## Appendix D

Short form SF-12 questionnaire [25]. Tick the appropriate box for each item.

1. In general, would you say your health is:

□ Excellent

□ Very good

□ Good

□ Fair

□ Poor

2. The following items are about activities you might do during a typical day. Does your health now limit you in these activities? If so, how much?

2a) Moderate activities, such as moving a table, pushing a vacuum cleaner, bowling, or playing golf

□ Yes, limited a lot

□ Yes, limited a little

□ No, not limited at all

2b) Climbing several flights of stairs

□ Yes, limited a lot

□ Yes, limited a little

□ No, not limited at all

3. During the past 4 weeks, have you had any of the following problems with your work or other regular daily activities as a result of your physical health?

3a.) Accomplished less than you would like

□ Yes

□ No

3b) Were limited in the kind of work or other activities

□ Yes

□ No

4. During the past 4 weeks, have you had any of the following problems with your work or other regular daily activities as a result of any emotional problems (such as feeling depressed or anxious)?

4a) Accomplished less than you would like

□ Yes

□ No

4b) Didn’t do work or other activities as carefully as usual.

□ Yes

□ No

5. During the past 4 weeks, how much did pain interfere with your normal work (including both work outside the home and housework)?

□ Not at all

□ A little bit

□ Moderately

□ Quite a bit

□ Extremely

6. These questions are about how you feel and how things have been with you during the past 4 weeks. For each question, please give the one answer that comes closest to the way you have been feeling. How much of the time during the past 4 weeks…

6a) Have you felt calm and peaceful?

□ All of the time

□ Most of the time

□ A good bit of the time

□ Some of the time

□ A little of the time

□ None of the time

6b) Did you have a lot of energy?

□ All of the time

□ Most of the time

□ A good bit of the time

□ Some of the time

□ A little of the time

□ None of the time

6c) Have you felt downhearted and blue?

□ All of the time

□ Most of the time

□ A good bit of the time

□ Some of the time

□ A little of the time

□ None of the time

7. During the past 4 weeks, how much of the time has your physical health or emotional problems interfered with your social activities (like visiting with friends, relatives, etc.)?

□ All of the time

□ Most of the time

□ A good bit of the time

□ Some of the time

□ A little of the time

□ None of the time

## Appendix E

Questionnaire about additional details on the subject and on the administration conditions of the PGWBI and SF-12 questionnaires.

- Age ________________________
- Gender __________________________
- Country __________________________
- Level of education __________________________
- Practiced agonistic sport (if any) __________________________
- IPC disability class for Paralympic athletes (if any, otherwise write NA = not applicable) ___________________________
- Living setting (e.g. either rural or urban area) ___________________
- Kind of disability (if any) ____________________________________________
- Mode of administration of the questionnaires (tick the appropriate circle)
  ○ self-administered via web submission
  ○ with the assistance of someone else via web submission (specify relationship with the helping person and entity of the help) ____________________________________________ ____________________________________________
  ○ self-administered in paper format
  ○ as a face-to-face interview with the help of a tutor or a linguistic mediator in paper format (specify the entity of the help) ____________________________________________ ____________________________________________
- Place of administration of the questionnaires (e.g. at home, in a clinic, at the venue of a sport competition event…) __________________________

## Abbreviations

CIP: Italian Paralympic Committee
FINP: Italian Federation for Paralympic Swimming
FISDIR: Italian Federation of Paralympic Sport for Intellectual and Relational Disabilities
FISPES: Italian Federation of Paralympic and Experimental Sports
FISPIC: Italian Federation of Paralympic Sport for visually impairment disabilities
FITARCO: Italian Federation of Archery
FITET: Italian Federation of Table Tennis
FIV: Italian Federation of Sailing
IPC: International Paralympic Committee
MCS-12: Mental Component Summary
PCS-12: Physical Component Summary
PGWBI: Psychological General Well-Being Index
SF-12: Short Form - 12 index
